# Preschool musicality is associated with school-age communication abilities through genes related to rhythmicity

**DOI:** 10.1038/s41539-025-00329-y

**Published:** 2025-06-13

**Authors:** Lucía de Hoyos, Ellen Verhoef, Aysu Okbay, Janne R. Vermeulen, Celeste Figaroa, Miriam Lense, Simon E. Fisher, Reyna L. Gordon, Beate St Pourcain

**Affiliations:** 1 Language and Genetics Department, Max Planck Institute for Psycholinguistics, Nijmegen, The Netherlands; 2 Department of Economics, School of Business and Economics, Vrije Universiteit Amsterdam, Amsterdam, The Netherlands; 3 Blair School of Music, Vanderbilt University, Nashville, TN USA; 4 Department of Otolaryngology - Head & Neck Surgery, Vanderbilt University Medical Center, Nashville, TN USA; 5 Department of Hearing and Speech Sciences, Vanderbilt University, Nashville, TN USA; 6 Donders Institute for Brain, Cognition and Behaviour, Radboud University, Nijmegen, The Netherlands; 7 Vanderbilt Genetics Institute, Vanderbilt University Medical Center, Nashville, TN USA; 8 MRC Integrative Epidemiology Unit, University of Bristol, Bristol, UK

**Keywords:** Human behaviour, Language, Learning and memory

## Abstract

Early-life abilities involved in perceiving, producing and engaging with music (musicality) may shape later (social) communication and language abilities. Here, we investigate phenotypic and genetic relationships linking musicality and communication abilities by studying information from preschool and school-aged children of the Avon Longitudinal Study of Parents and Children (*N* = 4169–6737 per measure, age 0.5–17 years). Using structural models, we identified relationships between latent musicality and speech- and cognition-related variables (r > 0.30). Consistently, polygenic scores for rhythmicity in adulthood (PGS_rhythmicity_) showed associations with preschool and school-age musicality (incremental-*Nagelkerke*-R^2^ = 0.006-0.011, *p* < 0.0025), as well as school-age communication and cognition-related measures (incremental-R^2^ = 0.04–1%, *p* < 0.0025). Studying the directionality of genetic effects using a mediation framework, we found evidence supporting a developmental pathway linking preschool musicality to school-age speech-/syntax-related abilities, as captured by PGS_rhythmicity_ (shared effect: β = 0.0051(SE = 0.0021), *p* = 0.015). Associations were found conditional on general cognition and genetically unrelated to educational attainment, suggesting robust developmental links between early musicality and later speech-related communication performance.

## Introduction

Communication is ubiquitous in human life, enabling us to interact with others, thus shaping communities and culture^1^. Communication requires speech-related (e.g., intelligibility and fluency of speech) and language-related (e.g., complexity of spoken grammar) skills, in addition to auditory processing. Communication within a social setting, known as social communication or pragmatic language, also involves social interaction-related skills^2^. In particular, social communication requires adequate language use, the adaptation of language to the listener or situation, and the adherence to conversational conventions (e.g., turn-taking or conversational rapport)^2^. Difficulties communicating with others are, therefore, also characteristic of many mental health problems and neurodevelopmental conditions^3^, including communication disorders^4^.

The acquisition of communication skills in typically developing children is shaped by social, linguistic, and cognitive factors^5,6^, and is also subject to other influences beyond traditionally defined general cognition. Early life musicality skills are an understudied but potentially important precursor of later social communication abilities. At the functional level, both musicality (i.e., the suite of abilities involved in perceiving, producing and engaging with music, including rhythmic abilities)^7^ and communication require acoustic, motor and perceptual skills, suggesting a shared biological basis^8,9^. Furthermore, many theories on the evolutionary and functional origins of music-making argue that musicality takes a key role in enhancing social bonding and facilitating cooperation within groups^10^.

Musicality skills, such as rhythmicity, emerge early in life; neonates can already detect the beat in music^11^. In infancy, rhythmicity promotes social communication between the caregivers and the child^12–14^. In preschool children, rhythmic abilities are related to phonological segmentation and phonological awareness, shaping language acquisition^15^. More generally, early-life music engagement has been associated with later-life communication aspects such as oral and written language^16,17^, as well as cognition and social skills^12^. Hence, improving our understanding of links between early-life musicality, including rhythmic skills, and later (social) communication abilities may help to gain insight into the aetiological mechanisms driving communication development in children.

Genetic factors partially explain individual differences in both communication and musicality. Twin studies of communication-related traits have reported high heritability estimates, ranging between 58% and 79%, for pragmatic language (i.e., the use of language in conversation and social situations) and communication skills^18^. Musicality-related traits are heritable, too, with twin studies reporting a heritability estimate of 50% for rhythm discrimination^19^. Recently, a genome-wide association study (GWAS) of self-reported beat synchronisation (i.e., a yes/no response to “Can you clap in time with a musical beat?”) was conducted in a large, well-powered sample of 606,825 individuals^20^. Investigating genetic variants present in at least 1% of the general population (indexed by single-nucleotide polymorphisms, SNPs), this study reported a SNP-based heritability of 13-16%^20^. The availability of this large GWAS^20^ enabled, for the first time, the study of genetic overlap between rhythmicity and other traits using genomic approaches, demonstrating associations with language^21,22^, mental health^23^ and other musicality measures^24,25^, mostly in adult populations.

Given phenotypic links between early musicality and speech-language skills^16,17^, we hypothesised that these relationships may extend to communication phenotypes and manifest in shared genetic links, especially during an early developmental window. Compared to phenotypic analyses, investigating genetic relationships can add specificity and robustness to phenotypic relationships^26^, especially when coupled with structural models that allow disentangling developmental processes. In particular, genetic analyses provided sophisticated tools to detect and adjust for confounding^27,28^. In this study, we aimed to control for genetic confounding of educational attainment (EA) influences, given that families with higher socioeconomic status (SES) may have easier access to music training^29^ but also foster improved (social) communication abilities^30,31^ leading to spurious association (confounding)^28^. Adopting a genomic approach has, thus, important implications for theoretical frameworks predicting links between musicality and language^16,32^.

To understand the relationship between early-life musicality and social communication, we studied unrelated children of European descent from the Avon Longitudinal Study of Parents and Children (ALSPAC)^33,34^ cohort with phenotypic and genetic data available. Here, we assess the phenotypic association between childhood musicality and school-age (social) communication abilities and model overarching multivariate structures. We use genetic tools, specifically those related to genetic influences underlying rhythmicity^20^, to assess whether genetic associations are consistent with an underlying developmental pathway from preschool musicality to school-age communication. Furthermore, we test whether the reported associations are robust to potential genetic confounding through EA, a proxy of SES with genetic contributions^35^.

## Results

### Study design

To better understand links between early-life musicality and (social) communication, we study phenotypic and genetic data from ALSPAC children (N = 4169 - 6737 per measure) by implementing a three-stage design.

Within the first stage, we identify and model the phenotypic links between preschool and school-age musicality as predictors and school-age (social) communication skills and cognition-related measures as outcomes. To do so, we (i) assess the evidence for phenotypic associations between predictor and outcome measures while controlling for genetic confounding using polygenic scores for EA (PGS_EA_)^28^. Subsequently, we (ii) model overarching relationships across associated measures using structural equation modelling (SEM) techniques.

Within the second stage, we assess the association between musicality and (social) communication using genetic instruments. Specifically, we study (iii) polygenic scores for rhythmicity (PGS_rhythmicity_), i.e., known genetic influences underlying musicality, to validate musicality measures in ALSPAC through genetic association, and identify genetic relationships with (social) communication outcomes that are consistent with shared underlying mechanisms, independent of genetic confounding (i.e., genes shared with EA).

Within the third stage of the study, we examine the direction of association between musicality and (social) communication. To this aim, we (iv) model phenotypic relationships between preschool musicality and school-age (social) communication, embedded in the first stage and linked to PGS_rhythmicity_ (*p* < 0.05) in the second stage, that are consistent with developmental processes, using SEM techniques. We focus on preschool musicality and school-age (social) communication to control for the directionality of the association, avoiding reverse causation bias (e.g., school-age communication abilities influencing preschool musicality). Eventually, we (5) study evidence for a developmental pathway linking preschool musicality to school-age communication through genes shared with rhythmicity (PGS_rhythmicity_) independent of genes shared with EA.

### Stage 1: Phenotypic associations between musicality and social communication

Our analyses comprised a total of 25 measures (Table 1) assessed during preschool (ages 6 months to 5 years, 5 measures) and school-age (ages 6 to 17 years, 20 measures) years. Musicality measures included two infant ALSPAC-specific nursery rhyme measures and three ALSPAC-specific musicality measures, which were assessed during preschool (age 5 years, 3 measures) and twice during school age (ages 6 and 7 years, 6 measures). School-age communication measures (ages 8 to 17 years) comprised four social communication measures from the Social and Communication Disorders Checklist (SCDC)^36^ across childhood and adolescence and eight measures of children’s communication assessed with the Children’s Communication Checklist (CCC)^37^. Additionally, capturing known links with language^21^, we studied school-age verbal cognition-related measures (age 9 years) linked to rhythmicity: verbal IQ score assessed with Wechsler Intelligence Scale for Children (WISC-III)^38^ and nonword repetition scores from the Nonword Repetition Test (NWRT)^39^.

**Table 1:** Descriptive information on predictor and outcome measures studied in the Avon Longitudinal Study of Parents and Children (ALSPAC)

*Predictors*	*N*	*Males / Females*	*Categories 1/2/3*	*Mean age in years (SD)*
***Preschool nursery rhymes***				
Nursery rhymes 0.5Y	6737	3426/3311	4437/838/1462	0.52 (0.05)
Nursery rhymes 1.5Y	6696	3434/3262	187/675/5834	1.51 (0.04)
***Preschool musicality phenotypes***				
Can sing at least 3 songs 5Y	6043	3098/2945	89/358/5596	4.78 (0.07)
Can hum a tune 5Y	6022	3091/2931	241/511/5270	4.78 (0.07)
Can clap to a beat 5Y	5968	3057/2911	403/1003/4562	4.78 (0.07)
***School-age musicality phenotypes***				
Can sing at least 3 songs 6Y	5676	2893/2783	43/241/5392	5.79 (0.08)
Can hum a tune 6Y	5674	2893/2781	108/278/5288	5.79 (0.08)
Can clap to a beat 6Y	5640	2872/2768	141/529/4970	5.79 (0.08)
Can sing at least 3 songs 7Y	5531	2815/2716	42/194/5295	6.78 (0.09)
Can hum a tune 7Y	5527	2817/2710	53/288/5186	6.78 (0.09)
Can clap to a beat 7Y	5498	2804/2694	68/432/4998	6.78 (0.09)

*Outcomes*	*N*	*Males / Females*	*Mean score (SD)*	*Mean age in years (SD)*
***School-age communication phenotypes (CCC)***				
Intelligibility and fluency 10Y	5645	2850/2795	35.31 (1.88)	9.64 (0.10)
Syntax 10Y	5628	2841/2787	31.84 (0.57)	9.64 (0.10)
Appropriate initiation 10Y	5623	2837/2786	26.75 (2.40)	9.64 (0.10)
Coherence 10Y	5628	2841/2787	34.85 (1.99)	9.64 (0.10)
Non-stereotyped conversation 10Y	5606	2829/2777	26.87 (2.47)	9.64 (0.10)
Use of conversational context 10Y	5549	2803/2746	29.84 (2.09)	9.64 (0.10)
Conversational rapport 10Y	5549	2802/2747	32.51 (1.95)	9.64 (0.10)
Pragmatic score 10Y	5524	2789/2735	150.86 (7.76)	9.64 (0.10)
***School-age social-communication phenotypes (SCDC)***				
SCDC score 8Y	5486	2814/2672	2.82 (3.70)	7.64 (0.11)
SCDC score 11Y	5383	2710/2673	2.34 (3.52)	10.71 (0.08)
SCDC score 14Y	5009	2501/2508	2.53 (3.57)	13.89 (0.11)
SCDC score 17Y	4169	2020/2149	2.84 (3.78)	16.84 (0.36)
***School-age cognition-related abilities***				
Nonword repetition 9Y (NWRT)	5398	2693/2705	7.26 (2.51)	8.62 (0.26)
Verbal IQ 9Y (WISC-III)	5378	2671/2707	107.98 (16.68)	8.62 (0.26)

Eleven predictor measures capturing preschool nursery rhymes and musicality, and school-age musicality were assessed in ALSPAC children aged 6 months to 7 years, using ALSPAC-specific instruments (see Methods). The table provides details on the total sample size, sex distribution, distribution across ordinal categories, and the mean age. Fourteen outcome measures capturing aspects of communication, social communication, working memory and cognition were assessed in ALSPAC children aged 8 to 17 years using standardised instruments (see Methods). The table includes information on the total sample size, sex distribution, mean scores, and mean age.

*SCDC* Social Communication Difficulties Checklist, *CCC* Children’s Communication Checklist, *SD* standard deviation.

**Fig. 1:**
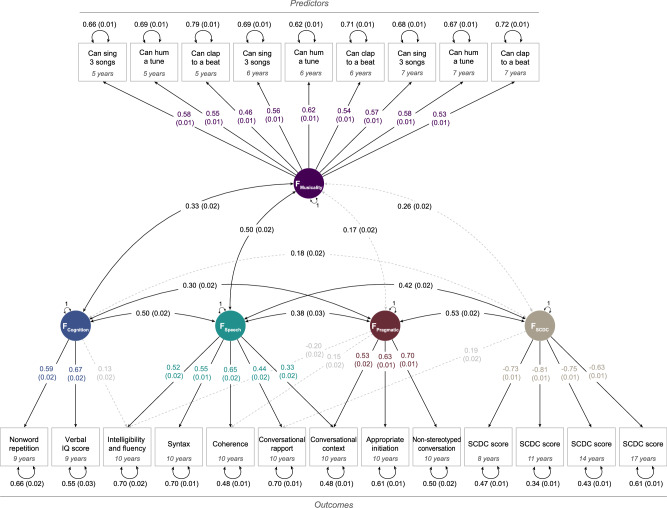
Structural model. Confirmatory factor analysis (CFA) model across preschool and school-age musicality predictor measures and school-age (social) communication and cognition-related outcome phenotypes (*N* = 5773). A detailed description of the measures can be found in Table 1. Standardised estimates are shown with their corresponding SEs, unstandardised estimates are available in Supplementary Table 4. Observed measures are represented by squares and latent variables by circles. Coloured single-headed arrows define meaningful factor loadings ( | λ | >0.30) and grey otherwise. Double-headed black arrows represent the variance of each phenotype and factor correlations. The CFA model provided an acceptable model fit (CFI = 0.90, TLI = 0.88, RMSEA = 0.049, SRMR = 0.034). Note that infant musicality measures (nursery rhymes) explained less than 1% of the variance in the outcome measures (Supplementary Fig. 1) and were not included in further analyses. SCDC Social Communication Difficulties Checklist, CCC Children’s Communication Checklist.

We examined the extent to which musicality measures (i.e., predictors) explain phenotypic variation in school-age communication and social communication (i.e., outcomes). After correcting for covariates (sex, age and the first ten ancestry-informative principal components, see Methods), preschool and school-age musicality measures explained up to 6% of the phenotypic variance in school-age communication and social communication measures (Supplementary Fig. 1, Supplementary Table 1) with associations passing the multiple-testing threshold (0.05/154 tests, *p* < 0.000324). In contrast, infant musicality measures (nursery rhymes) explained less than 1% of the variance in the outcome measures (Supplementary Fig. 1) and were, thus, not taken forward in subsequent analysis. To control for mechanisms of genetic confounding, we studied whether the association between musicality and communication measures is attenuated when adjusted for polygenic scores for EA (PGS_EA._), using a similar approach to previous studies^28^. We confirmed the robustness of the findings (Supplementary Table 1), observing little change in the strength of the associations (Supplementary Fig. 2 vs. Fig. 1).

Given evidence of association between predictor and outcome measures, we explored their overarching relationships through multivariate modelling at the phenotypic level using a data-driven modelling approach (see Methods)^40^. We identified a correlated 5-factor model (Fig. 1, Supplementary Fig. 3, Supplementary Table 2) with good fit indices (CFI = 0.90, TLI = 0.88, RMSEA = 0.049, SRMR = 0.034). Focusing on measures with meaningful standardised factor loadings ( | λ | > 0.3)^41^, the five factors explained phenotypic variation across musicality measures (F_musicality_), cognition (F_cognition_), speech-related aspects of communication (F_speech_), pragmatic-related aspects of communication (F_pragmatic_) and social communication (F_SCDC_).

Variation across all preschool and school-age musicality measures was captured by F_Musicality_, with the highest loading on humming a tune at 6 years (λ = 0.62(SE = 0.01)). F_cognition_ reflected phenotypic variation across working memory (λ = 0.59(SE = 0.02)) and cognition at 9 years (λ = 0.67(SE = 0.02)). Variation across communication measures, assessed with the CCC, was explained by two different factors underlying speech-related (F_speech_) and pragmatic-related (F_pragmatic_) aspects of communication, with the highest loadings for Coherence (F_speech_ λ = 0.65(SE = 0.02)) and Non-stereotyped conversation (F_pragmatic_ λ = 0.70(SE = 0.01)), respectively. Variation across social communication measures from the SCDC was accounted for by F_SCDC_ with the largest loading for the SCDC score at 11 years (λ = -0.81(SE = 0.01)). Focusing on meaningful factor correlations ( | r | >0.3), we observed associations of F_musicality_ with F_cognition_ (r = 0.33(SE = 0.02)) and F_speech_ (r = 0.50(SE = 0.02)) but not with factors underlying more social aspects of communication (F_pragmatic_ and F_SCDC_). Note that the CCC pragmatic composite score, composed of four subscales of the CCC^37^, was excluded from the multivariate analysis to avoid collinearity.

### Stage 2: Genetic association analysis

Next, we hypothesised that true developmental processes linking early-life musicality to (social) communication will involve shared genetic links, as captured by a genetic predisposition to rhythmicity (i.e., PGS_rhythmicity_).

**Fig. 2:**
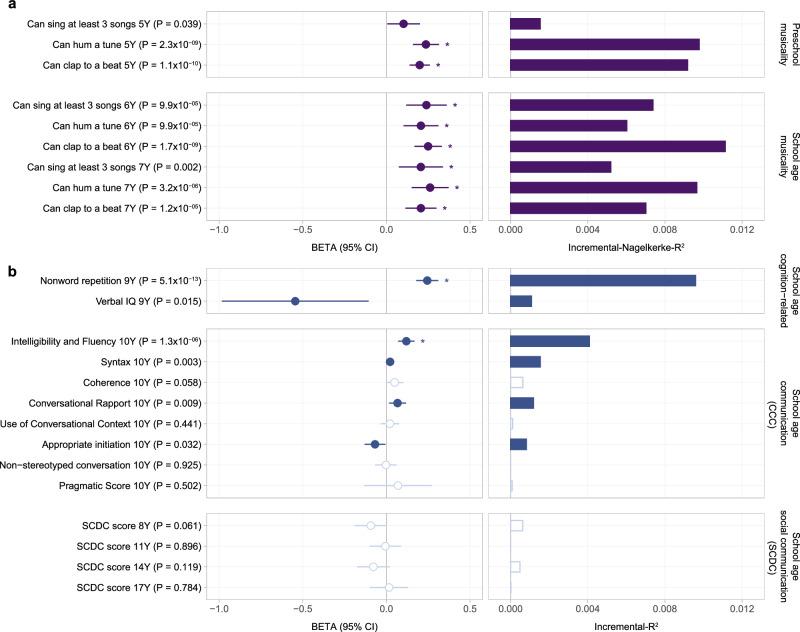
Polygenic scoring analyses with polygenic load for rhythmicity. **a** Association of PGS_rhythmicity_ with preschool and school-age musicality predictor measures using proportional odds logistic regression (*N* = 5483–6028). Beta estimates are shown as circles with their corresponding 95% confidence intervals. The goodness of fit is shown as incremental *Nagelkerke*-R^2^. Note that infant musicality measures (nursery rhymes) explained less than 1% of the variance in the outcome measures (Supplementary Fig. 1) and were not included in further analyses. **b** Association of PGS_rhythmicity_ with school-age communication, social communication and verbal-cognition outcome measures using linear regression (*N* = 4169–5645). Beta estimates are shown as circles with their corresponding 95% confidence intervals. The variance explained for each phenotype is shown as bars and expressed as incremental R^2^. Filled circles/bars and empty circles/bars represent phenotypes with an association with PGS_rhythmicity_ of *p* < 0.05 and *p* ≥ 0.05, respectively. If a phenotype passed the multiple-testing threshold of 0.0025, this was indicated with an asterisk. A table with estimates is shown in Supplementary Table 3. SCDC, Social Communication Difficulties Checklist; CCC, Children’s Communication Checklist.

First, we genetically validated the selected musicality measures in ALSPAC through association with PGS_rhythmicity_ (*p* < 0.05, Fig. 2a, Supplementary Table 3). All associations except for singing songs at 5 years passed the multiple-testing threshold (*p* < 0.0025, incremental *Nagelkerke*-R^2^: 0.006–0.011). The identified genetic overlap with musicality-related measures confirms previous findings^20^ and extends known associations between PGS_rhythmicity_ and musicality to an earlier developmental window.

Next, we screened the 14 school-age outcomes for polygenic overlap with rhythmicity. We found evidence for two associations passing the multiple-testing threshold (*p* < 0.0025, incremental R^2^: 0.004–0.01, Fig. 2b) and four associations at the nominal level (*p* < 0.05, Fig. 2b, Supplementary Table 3). Genetic associations included four CCC subscales at 10 years: intelligibility and fluency (β = 0.12(SE = 0.025), *p* = 1.3 × 10^−6^), syntax (β = 0.022(SE = 0.0075), *p* = 3.2 × 10^−3^), appropriate initiation (β = −0.068(SE = 0.032), *p* = 3.2 × 10^-2^) and conversational rapport (β = 0.067(SE = 0.026), *p* = 9.3 × 10^−3^); and two school-age verbal cognition-related measures: nonword repetition (β = 0.24(SE = 0.034), *p* = 5.1 × 10^−^^13^), and verbal IQ (β = -0.55(SE = 0.22), *p* = 1.5 × 10^-2^). The overlap between rhythmicity and nonword repetition confirms, using individual-level genetic data, previously reported findings based on summary statistics^21^.

As genetic factors underlying rhythmicity and EA are marginally interrelated^20^, we performed additional analyses to rule out that genetic confounding via EA contributes to genetic effects shared with PGS_rhythmicity_^42^. Specifically, we applied GWAS-by-subtraction techniques^27^ to create PGS_rhythmicity-EA_ (Methods), thereby removing EA genetic variance from rhythmicity as captured by GWAS statistics, and repeated the analyses described above. We identified a similar pattern of genetic overlap across the studied measures, especially for associations passing the multiple-testing threshold (Supplementary Fig. 4, Supplementary Table 4), suggesting limited confounding through EA.

Together, these findings confirm that PGS_rhythmicity_ explains variation in all studied musicality measures, including preschool and school-age musicality. Importantly, they also suggest that genetic influences underlying rhythmicity contribute to school-age cognition, working memory and speech-related communication abilities, consistent with overarching developmental processes.

### Stage 3: Disentangling developmental pathways between preschool musicality and school-age communication using genetic information

To investigate whether the overlap of PGS_rhythmicity_ with both musicality and communication also captures phenotypic links between musicality and communication that are consistent with developmental processes, we modelled multivariate structures across PGS_rhythmicity_-associated measures (PGS_rhythmicity_ *p* < 0.05, Fig. 2). To avoid reverse causation bias due to schooling^43^, we focus on preschool musicality (age 5 years), preceding school-age communication (ages 9 and 10 years), excluding musicality measures at later ages.

**Fig. 3:**
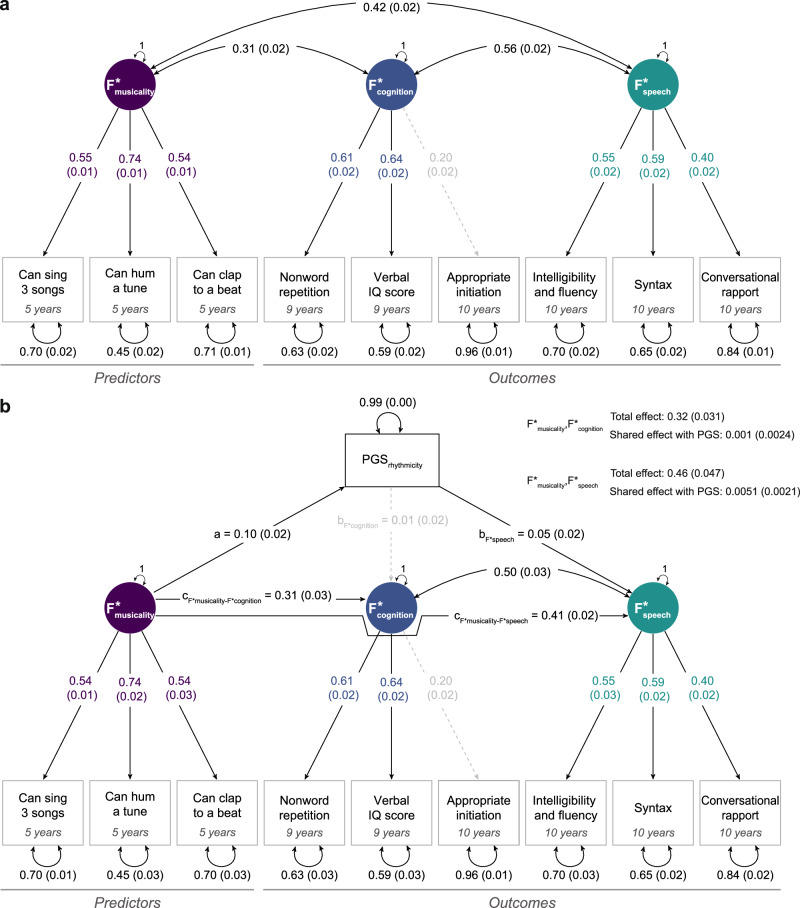
Developmental pathways of preschool musicality with school-age cognition-related skills and school-age communication skills. **a** Confirmatory factor analysis (CFA) model of preschool musicality predictor measures and school-age communication and cognition-related outcome measures sharing variation with PGS_rhythmicity_ (*N* = 5873). Standardised estimates are shown with their corresponding SEs, unstandardised estimates are shown in Supplementary Table 5. Observed measures are represented by squares and latent variables by circles. Coloured single-headed arrows define factor loadings with p ≤ 0.05. Double-headed black arrows represent the variance of each phenotype and factor correlations. The CFA model provided an optimal model fit (CFI = 0.95, TLI = 0.93, RMSEA = 0.042, SRMR = 0.028). **b** Genetic characterisation of phenotypic relationships between F*_musicality_ on F*_cognition_ and F*_speech_ explained by shared links with PGS_rhythmicity_ (*N* = 5873). The shared genetic effect between F*_musicality_ and F*_speech_, as captured by PGS_rhythmicity_, is estimated as a*b_F3_ and the total effect between F*_musicality_ and F*_speech_ as a*b_F*speech_ + c_F*musicality-F*speech_. The shared genetic effect between F*_musicality_ and F*_cognition_, as captured by PGS_rhythmicity_, is estimated as a* b_F*cognition_ and the total effect as a* b_F*cognition_ + c_F*musicality-F*cognition_. Standardised estimates are shown with their corresponding SEs, unstandardised estimates and shared effects with PGS_rhythmicity_ are shown in Supplementary Table 6. Observed measures are represented by squares and latent variables by circles. Coloured single-headed arrows define factor loadings with *p* ≤ 0.05. Double-headed black arrows represent the variance of each phenotype and factor correlations. Grey dotted and black solid single-headed arrows define relationships between factors and with PGS_rhythmicity_ with *p* > 0.05 and *p* ≤ 0.05, respectively.

Relationships across PGS_rhythmicity_-associated measures were modelled adopting a data-driven approach (see Methods)^40^. The final SEM model included the following PGS_rhythmicity_-associated measures: three preschool musicality measures, four school-age communication measures and two school-age language/cognition measures. We identified a correlated three-factor model, which showed a good model fit (CFI = 0.95, TLI = 0.93, RMSEA = 0.042, SRMR = 0.028, Fig. 3, Supplementary Fig. 5, Supplementary Table 5), with structural similarities compared to the phenotypic model (Fig. 1). A preschool musicality factor (F*_musicality_) described phenotypic variation of preschool ability to sing 3 songs (λ = 0.55(SE = 0.01)), hum a tune (λ = 0.74(SE = 0.01)) and clap to a beat (λ = 0.54(SE = 0.01)) at 5 years. The school-age verbal cognition factor (F*_cognition_) captured variation within nonword repetition (λ = 0.61(SE = 0.02)) and verbal IQ (λ = 0.64(SE = 0.02)) at 9 years as well as inappropriate initiation at 10 years (λ = 0.20(SE = 0.02)). A school-age communication factor (F*_speech_) explained variation across three of the CCC subscales at 10 years: intelligibility and fluency (λ = 0.55(SE = 0.02)), syntax (λ = 0.59(SE = 0.02)) and conversational rapport (λ = 0.40(SE = 0.02)). Inter-factor correlations with F*_musicality_ were modest (r_F*musicality,F*cognition_ = 0.31(SE = 0.02), r_F*musicality,F*speech_ = 0.42(SE = 0.02)), while the correlation between F*_cognition_ and F*_speech_ was moderate (r_F*cognition,F*speech_ = 0.56(SE = 0.02)). Thus, PGS_rhythmicity_-associated measures are captured by three overarching phenotypic dimensions that are embedded within the phenotypic SEM (Fig. 3) and, through association with PGS_rhythmicity_, may reflect shared aetiological processes.

Next, following the confirmation of phenotypic structure across these PGS_rhythmicity_-associated measures, we mapped genetic links with PGS_rhythmicity_ to the identified factor structure to seek evidence for a developmental pathway linking preschool musicality to school-age communication through genes shared with rhythmicity. Adopting an analytical framework analogous to mediation analysis (Methods), we studied the directionality of effects and dissected the factorial relationships between preschool F*_musicality_ (predictor) and school-age F*_cognition_ and F*_speech_ (outcomes), modelling association effects shared and not shared with PGS_rhythmicity_ (Fig. 3b, Supplementary Table 6). After adding PGS_rhythmicity_ to the CFA model structure, the model fitted the data similarly well (CFI = 0.93, TLI = 0.90, RMSEA = 0.044, SRMR = 0.029), the factor structure remained otherwise unchanged, and factor loadings were virtually identical (Fig. 3a vs Fig. 3b).

Our analyses demonstrate that the association between preschool musicality skills (F*_musicality_) and school-age communication skills (F*_speech_) (total effect: β = 0.46 (SE = 0.047), *p* = 1.59 × 10^−9^) is partially accounted for by PGS_rhythmicity_ (shared effect: β = 0.0051 (SE = 0.0021), *p* = 0.015, Supplementary Table 6). In contrast, there was little evidence that the variation shared with PGS_rhythmicity_ contributes to the association between preschool musicality (F*_musicality_) and school-age verbal cognition (F*_cognition_) (shared effect: β = 0.001 (SE = 0.002), *p* = 0.71; total effect: β = 0.32(SE = 0.031), *p* = 2.35 × 10^−11^). Thus, while there are strong links between F*_musicality_ and F*_cognition_, they are not accounted for by PGS_rhythmicity_. All factor structures and association patterns remained robust when removing genetic confounding through EA by studying PGS_rhythmicity-EA_ (Supplementary Fig. 6, Supplementary Table 7).

Thus, links between latent preschool musicality (F*_musicality_) and school-age communication (F*_speech_), but not verbal cognition (F*_cognition_), are partially attributable to genetic variation underlying rhythmicity (beat synchronisation) and robust to genetic confounding (i.e., independent of genetic variation contributing to EA). Disentangling overarching phenotypic patterns observed in Stage 1, these association patterns suggest that preschool musicality is robustly linked to school-age communication-related abilities, but not school-age cognition, as partially captured by polygenic load contributing to rhythmicity in large genome-wide studies^20^.

## Discussion

Investigating a UK population-based sample of unrelated children, this study showed that (i) musicality measures can predict school-age communication, social communication and verbal-cognition outcomes, (ii) preschool musicality and school-age communication abilities share genetic links with rhythmicity, and (iii) genetic association patterns are consistent with a developmental pathway between preschool musicality and school-age communication. These findings are in support of aetiological mechanisms, where preschool musicality, as captured by genes underlying rhythmicity (i.e., PGS_rhythmicity_), contributes to children’s school-age communication abilities during mid-childhood, above and beyond influences underlying general cognition and robust to genetic confounding.

Our phenotypic analysis revealed factor structures linking musicality (ages 5-7 years) modestly to cognition (age 9 years) and moderately to speech-related aspects of communication (age 10 years, captured by CCC subscales). Associated musicality scores, including singing songs, clapping to a beat and humming a tune at ages 5 to 7 years, as captured by the musicality factor, shared throughout genetic influences contributing to rhythmicity in adulthood. Thus, the association with PGS_rhythmicity_ validates ALSPAC-specific musicality measures and strengthens previously reported evidence for links across PGS_rhythmicity_ and objective tests of rhythm ability^25^. Furthermore, given that the GWAS on beat synchronisation was conducted on an adult sample (mean age = 52.09)^20^, the association of PGS_rhythmicity_ with early musicality measures demonstrates that the genetic underpinnings of being able to clap to a beat in adulthood are transferable across the lifespan. Among the musicality-associated outcome measures, we found links between PGS_rhythmicity_ and language- and communication-related phenotypes. Importantly, the direction of genetic effects was consistent with developmental processes linking preschool musicality to school-age communication (but not cognition) and was partially captured by PGS_rhythmicity_. These findings converge with previous studies reporting a phenotypic association between spoken language and rhythmic abilities in children^16^, extending them to speech-related communication abilities. Consistently, rhythm perception and production are known predictors of phonological awareness, while melody perception has been linked to grammar acquisition^44^. Thus, these results are consistent with two theoretical frameworks discussing the potential role of rhythmicity in language- and communication-related traits: *Musical Abilities, Pleiotropy, Language, and Environment* (MAPLE) framework^16^, which states that musicality and language share genetic variation and therefore musicality and language are interrelated, and the *Atypical Rhythm Risk Hypothesis*^32^, which proposes that rhythm impairment is associated with delays in speech/language abilities.

While PGS analyses can only account for a small proportion of phenotypic variance, they can link association patterns to known genetic influences underlying musicality. Findings gained further support as the latent dimensions of PGS_rhythmicity_-associated measures were corroborated by the observed phenotypic structures that were unconditional on an association with PGS_rhythmicity_. Links between preschool musicality and school-age speech-related communication abilities were independent of EA-related influences, observed conditional on cognition and the direction of genetic effects was confirmed with a mediation-based framework exploiting the temporal order of phenotypes. Thus, our findings are robust and unlikely to be the consequence of reverse causation bias. Assuming true underlying developmental processes, links between musicality and communication may arise due to mediated pleiotropy^45^, where genetic influences related to musicality may become indirectly associated with communication phenotypes. Impaired rhythmic skills and timing have been highlighted as an early predictor of atypical developmental cascades^46^ that may contribute to later language and communication difficulties^32^. Musical, and in particular rhythm, training during early childhood has been implemented as an intervention to improve school-age speech and syntactic language abilities^47,48^. Thus, our findings provide further support implicating musicality in developmental processes underlying communication, above and beyond general cognition.

In contrast, the musicality factor was only weakly correlated with the pragmatic and social communication factors, and the underlying measures showed no or only nominal evidence for association with PGS_rhythmicity_. In particular, the phenotypic correlation between latent musicality and pragmatic abilities differed from links between latent musicality and speech- and cognition-related outcomes, with non-overlapping 95% confidence intervals. Given comparable power and cohort design (see below), aetiological links between preschool musicality and pragmatic/social communication skills may be distinct from processes shaping speech, working memory and cognition. In addition, the developmental processes linking musicality to cognition-related traits may be different from those influencing speech-related traits. While the school-age cognition-related factor was correlated with the musicality factor and associated with PGS_rhythmicity_, the shared effects with PGS_rhythmicity_ did not explain links with musicality. However, larger samples with broader phenotype definitions and stronger genetic instruments are required to identify differences in effect based on non-overlapping 95% confidence intervals.

Our study has multiple strengths. First, we introduced a multivariate modelling framework which combines PGS analyses with mediation and SEM techniques, disentangling phenotypic dimensions related to musicality, working memory, verbal cognition, speech and pragmatic and social communication. This approach allows us to assess the robustness and directionality of developmental relationships between latent variables using genetic tools, while combining and embedding PGS_rhythmicity_-related associations into latent phenotypic structures that benefit from enhanced phenotypic validity and reduced measurement error^49,50^. Second, by drawing power from the large, independent rhythmicity GWAS analysis^20^, we validated ALSPAC-specific musicality measures and increased the evidence for an association between preschool musicality and school-age speech-related communication abilities using a related phenotype. Third, combining PGS_rhythmicity_-related associations into latent phenotypic structures enhanced the power of the study, given the small proportion of variance explained by the PGS_rhythmicity_, and enabled a direct comparison with overarching phenotypic SEM structures, unconditionally on association with PGS_rhythmicity_. Fourth, we assessed the consistency of the association with developmental processes by controlling for reverse causation bias (studying phenotypes in a temporal order) and by assessing the directionality of the association that captures shared variation with PGS_rhythmicity_ (using a mediation framework^42^). Fifth, we confirmed the robustness of phenotypic links between preschool musicality and speech-related aspects of communication at school age to genetic confounding driven by genetic variation underlying EA.

Nonetheless, our work has several limitations. First, the ALSPAC cohort, like other longitudinal cohorts, is affected by loss to follow-up, and, consequently, findings might also be affected by attrition bias^51,52^. However, as presented findings are independent of genetic factors contributing to EA in the studied children, such bias is less likely. Second, the presented PGS association analyses need replication in an independent population-based cohort, which is challenging given the scarcity of longitudinal birth cohorts with genomic data and both (social) communication and musicality phenotypes. Future research should consider the longitudinal collection of a wide range of musicality and communication phenotypes in powerful multi-ancestry samples to enable the replication of results as well as to refine and characterise the biological processes underlying the reported findings. Third, the proportion of variance explained by PGS_rhythmicity_ was small (incremental R^2^: 0.004-0.01, incremental *Nagelkerke*-R^2^: 0.006–0.011). However, this proportion is in line with other studies of complex traits^53^, including the original publication (incremental R^2^ = 0.02)^20^, and it will potentially increase when studying larger GWAS^53^. Fourth, phenotypic and genetic association analyses were conducted using untransformed phenotypes, while SEM analyses were carried out with transformed (covariate-adjusted) measures to ease the computational burden. This is unlikely to affect the presented association patterns, as findings were highly consistent across stages, although we cannot fully exclude transformation bias. Fifth, the association between musicality and social communication phenotypes, which were only weakly correlated in this study, may become detectable when studying a broader set of social and musicality phenotypes. Given the extensive scope of human musicality^7^, there might be links between social communication and musicality beyond rhythm production, humming and singing. Likewise, other aspects of human social behaviour (such as social engagement or social reward, not included in this study) may reveal different association patterns. However, power is an unlikely explanation for the difference in association effects observed for speech-related versus social communication phenotypes in this study, as measures were collected with a comparable study design and, for CCC scores, also questionnaire design. Similarly, differences in genetic association effects are unlikely to reflect power differences. Assuming a h^2^_SNP_ of 0.05 for rhythmicity (on the observed scale)^20^ and of 0.15 for SCDC scores at 11 years old^54^, and a genetic correlation of 0.5 between traits, we have about 80% power to detect a polygenic effect (corresponding to an R^2^ = 0.0027) at an alpha level of 0.05^55,56^. Therefore, differences in association profiles of social-communication phenotypes may pinpoint to differences in aetiological mechanisms. Ongoing GWAS efforts within the Musicality Genomics Consortium (https://www.mcg.uva.nl/musicgens/) are aiming to conduct large GWAS on other musicality domains and could help disentangling the phenotypic relationships between musicality and (social) communication further.

In conclusion, we show that preschool musicality and school-age speech- and syntax-related communication abilities are interlinked, above and beyond general cognition. Association patterns are consistent with developmental processes, robust to genetic confounding and linked to known genetic influences underlying rhythmicity. Consequently, preschool musicality involving rhythmic skills may represent a precursor of school-age speech-related communication skills.

## Methods

### Sample description

ALSPAC is a population-based longitudinal pregnancy-ascertained birth cohort from the United Kingdom^33,34^ (estimated birth date 1991-1992, Supplementary Note 1). Ethical approval for the study was obtained from the ALSPAC Ethics and Law Committee (ALEC; IRB 00003312) and the Local Research Ethics Committees (Bristol and Weston Health Authority (E1808, 28^th^ November 1989); Southmead Health Authority (49/89, 5th April 1990); Frenchay Health Authority, (90/8, 28th June 1990)). Consent for biological samples has been collected following the Human Tissue Act (2004). Informed consent for the use of data collected via questionnaires and clinics was obtained from participants following the recommendations of the ALSPAC Ethics and Law Committee at the time. Further details on ethical approvals are available at http://www.bristol.ac.uk/alspac/researchers/research-ethics/.

### Genetic information

Genotyping was performed using the Illumina HumanHap550 quad chip. Standard genetic quality control checks at the SNP and individual level were carried out in PLINK^57^ (Supplementary Note 2). After quality control, our study comprised 8226 unrelated children (51% males) of European genetic ancestry and 465,740 SNPs with high-quality direct genotyping data, which were imputed to the Haplotype Reference Consortium reference panel (version r1.1) using the Sanger Imputation Server (https://imputation.sanger.ac.uk). Following standard guidelines, ancestry-informative principal components were computed for ALSPAC children based on genetic data and were included in subsequent analysis as a covariate to control for population stratification^58^.

### Phenotype information

Parent-reported measures of nursery rhymes, musicality, communication, social communication, and verbal-cognition were assessed in ALSPAC children (*N* = 4169–6737 per measure, Table 1). The study website contains details of all the available data through a fully searchable data dictionary and variable search tool (http://www.bristol.ac.uk/alspac/researchers/our-data).

For this study, we define a developmental preschool window (0-5 years) before children enter key stages of school curricula in the UK (i.e., preschool and reception years), compared to a developmental school-age window (>5 years), where children enter educational key stages (i.e., year 1 and above). Once children start schooling, many predictors of language-related phenotypes, such as phonological awareness, become reciprocally shaped by reading experience and may no longer reflect underlying aetiological mechanisms^43^.

### Phenotypic measures: predictors

Child’s participation in rhythmic play during preschool (0.5 and 1.5 years) was assessed based on parent reports of child’s engagement in nursery rhymes and clapping games (e.g., pat-a-cake). Responses were recorded using a 3-point Likert scale (“Yes does often”; “Has only done once or twice”; “Has not done yet”) and were reverse coded so that higher scores indicated greater ability to engage in nursery rhymes.

Musicality-related abilities were assessed during preschool (5 years) and school-age (6-7 years) through parent reports of each child’s ability to sing at least three songs, hum a tune, and clap in time with a musical beat. These items were rated on a 3-point Likert scale (“Yes, can do well”; “Yes, but not well”; “Has not yet done”) and scores were reverse coded so that higher values reflect stronger musicality ability.

### Phenotypic measures: outcomes

School-age social communication was evaluated at ages 8, 11,14, and 17 years using **t**he Social and Communication Disorders Checklist (SCDC)^36^, a 12-item questionnaire completed by parents. Each item measured aspects of the child’s social interaction and communication skills and was rated on a 3-point Likert scale (“Not true”; “Quite/sometimes true”; “Very/often true”). In this study, we used the SCDC total score, calculated as the sum of all 12 items. This demonstrated high internal consistency (Cronbach’s Alpha=0.86) and strong test-retest reliability (range = 0.84–0.93)^36^.

At 10 years old, school-age communication abilities were assessed in ALSPAC children using the Children’s Communication Checklist (CCC)^37^, a 70-item parent-reported questionnaire that consists of seven subscales: (A) intelligibility and fluency, (B) syntax, (C) inappropriate initiation, (D) coherence, (E) stereotyped conversation, (F) use of conversational context, and (G) conversational rapport. Items were rated on a 3-point Likert scale (“Certainly true”; “Somewhat true”; “Not true”). A pragmatic communication score was derived from subscales C to G. CCC subscales have a moderate/high internal consistency (0.62-0.83) and high reliability (0.74–0.87)^37^. Within this study, we refer to subscales C and E as appropriate initiation and non-stereotyped conversation, respectively, to align with the scoring direction of where higher values reflect less communication difficulties.

We included school-age measures of children’s verbal abilities (verbal IQ) and phonological working memory (nonword repetition). Both scores were assessed at age 9 years and measured with an abbreviated form of the Wechsler Intelligence Scale for Children (WISC-III)^38^ and the Nonword Repetition Test (NWRT)^39^, respectively (Supplementary Note 3).

### Phenotypic associations between musicality and social communication

To assess the relationship between musicality measures (predictors) and school-age outcomes, we conducted single linear regression analyses in which we compared a reduced model, including the covariates only, to a full model, which includes covariates and the predictor measure. The included covariates were age, sex and the first ten ancestry-informative principal components. For each pair of predictor-outcome measures, we assessed the increase in R^2^ after adding the predictor measure (i.e. incremental R^2^), and the p-values were obtained using an ANOVA test between the reduced and the full model.

To control for genetic confounding with EA, we computed PGS_EA_^35^ (see below detailed explanation of PGS computation, Supplementary Note 4) and added it to the covariates in the regression model. Therefore, the reduced model contains the covariates and PGS_EA,_ whereas in the full model, the outcome is regressed on the covariates, PGS_EA_ and the predictor measure. As before, we assessed the explanatory value of the predictor in terms of incremental R^2^ by comparing the reduced and the full model and *p*-values were obtained using an ANOVA test between the reduced and the full model.

### Multivariate modelling

To conduct multivariate analysis across a set of traits, we employed a data-driven approach (Supplementary Note 5). First, we estimated the number of factors using principal component analysis based on the phenotypic correlation matrix, studying all available data. Second, we split the sample into two random halves, matched for sex and missingness patterns. Third, we fitted an exploratory factor analysis (EFA) to the first half of the sample (*N* = 3048). To capture relationships across predictors and outcomes while maintaining the developmental nature of the phenotypes, two different EFA blocks were defined, one for predictors and another one for the outcomes, while allowing for correlation across the factors (exploratory-SEM model^59^). To approximate the EFA factor structure, we retained standardised EFA factor loadings (λ), capturing at least 1% of the phenotypic variation ( | λ | >0.1). Fourth, the identified EFA structure was used to inform subsequent confirmatory factor analysis (CFA), fitted in the other half of the sample (*N* = 3053). CFA model fit was assessed using the comparative fit index (CFI), the Tucker–Lewis index (TLI), the Root Mean Square Error of Approximation (RMSEA) and the Standardised Root Mean Square Residual (SRMR) parameters (Supplementary Note 5). The EFA and CFA models were fitted using both orthogonal (varimax) and oblique (oblimin) rotation in *lavaan* (R::lavaan,v0.6-14)^60^. Fifth, we used the structure of the best-fitting CFA model to inform the CFA on the full sample, where standard errors were computed using 1000 bootstraps.

To control for covariate effects, EFA and CFA were conducted with transformed phenotypes, adjusting for age, sex and the first ten ancestry-informative principal components (Supplementary Note 6). Note that joint analyses of multiple phenotypes within a factor analysis framework do not require adjusting for multiple testing.

### Polygenic score analysis

PGS are a weighted sum of alleles associated with a trait of interest that are carried by an individual^61^. A “discovery” GWAS of the trait of interest (here rhythmicity) is used to extract the respective weights for each genetic variant. The “target” sample (here ALSPAC) is used to calculate, for each individual (here ALSPAC children), their genetic propensity score for the trait of interest based on the genetic variants they carry and to run, across all individuals, an association analysis between such score and a set of phenotypes.

As a discovery sample, in this study, we used GWAS summary statistics on self-reported beat synchronisation (hereafter referred to as rhythmicity)^20^ from 23andMe Inc., including 606,825 individuals of European descent (91.5% controls, 64% females). Note that the original authors validated this self-reported rhythmicity measure using PGS and an independent experiment^20^, and, additionally, this measure showed overlap with well-validated music tests in follow-up studies^25^. As the target sample, we used imputed genotype data from ALSPAC children. Specifically, we extracted a set of common (minor allele frequency>1%) and well-imputed (imputation INFO score>0.8) HapMap 3 SNPs (*N* = 985,350).

PGS were computed using PRS-CS^62^, a method that applies a continuous-shrinkage parameter to adjust the effect sizes of the genetic markers for local linkage disequilibrium (LD) patterns. To do so, PRS-CS uses an external LD reference panel of European individuals from the 1000 genomes project^62^. Using these re-estimated effect sizes, PGS were generated with PLINK^57^ and, subsequently, Z-standardised. Note that PRS-CS does not require the selection of a *p*-value, as required for clumping and thresholding methods^62^.

To test for the association of ALSPAC phenotypes with the PGS, we used linear regression (continuous phenotypes) or proportional odds logistic regression (ordinal phenotypes) models. Regression analyses were corrected for age, sex and the first ten ancestry-informative principal components. The variation explained by the PGS_rhythmicity_ was expressed in terms of incremental R^2^ for continuous traits and, in analogy, incremental *Nagelkerke-*R^2^ for ordinal traits (Supplementary Note 4).

To adjust for multiple testing, we computed the effective number of phenotypes across the initial set of 25 phenotypes. We carried out matrix Spectral Decomposition^63^, which identifies the number of independent phenotypes based on phenotypic correlations. This yielded a multiple-testing threshold of 0.0025 (0.05/20 estimated independent phenotypes).

### Genetic characterisation of phenotypic structures using PGS

We tested whether the correlation between phenotypic factors was partially attributable to shared variation with PGS_rhythmicity_ while keeping the identified CFA factor structure otherwise fixed. Specifically, we incorporated PGS_rhythmicity_ into the CFA model structure by applying a framework analogous to mediation analysis (Supplementary Fig. 7)^64^. Note that the indirect effect within a mediation framework will estimate the shared effect between two phenotypic factors as captured by PGS_rhythmicity_.

### GWAS-by-subtraction

To control for genetic confounding in the second and third stages of the study, we generated rhythmicity GWAS^20^ summary statistics, excluding genetic effects shared with the EA GWAS summary statistics^35^ (rhythmicity-EA) by using a GWAS-by-subtraction framework^27^. GWAS-by-subtraction is an approach that fits a Cholesky model in genomic structural equation modelling^65^ (R::genomicSEM, v0.0.5) based on GWAS summary statistics from two traits, in our case, rhythmicity and EA (Supplementary Fig. 8). Within the EA GWAS, EA was measured in years of education adjusted for sex, year of birth, their interaction and genetic quality control measures^35^. To avoid sample overlap, ALSPAC and 23andMe individuals were excluded from EA summary statistics (personal communication with A. Okbay)^35^. Using the derived GWAS_rhythmicity-EA_, we created PGS_rhythmicity-EA_. Following guidelines by the original authors^27^, the effective sample size of the GWAS_rhythmicity-EA_ was estimated as *N* = 143,800.

## Supplementary information

Supplementary Information

Supplementary Tables

## Data Availability

The data used are available through a fully searchable data dictionary (http://www.bristol.ac.uk/alspac/researchers/our-data/). Access to ALSPAC data can be obtained as described within the ALSPAC data access policy (http://www.bristol.ac.uk/alspac/researchers/access/).

## Code availability

This study used openly available software. Specifically, PLINK (PLINK v1.9, https://www.cog-genomics.org/plink/1.9/), PRScs (https://github.com/getian107/PRScs). Analyses were mainly performed in R (version 4.1.1) using the following R packages: lavaan (v0.6-14), genomicSEM (v0.0.5), rcompanion (v2.4.30), dplyr (v1.0.8), nFactors (v2.4.1), Matrix (v1.4-1). Scripts used in this study are available in GitLab (https://gitlab.gwdg.de/pghc/genetic-and-phenotypic-links-between-musicality-and-communication).
